# Membrane binding of the neuronal calcium sensor recoverin – modulatory role of the charged carboxy-terminus

**DOI:** 10.1186/1471-2091-8-24

**Published:** 2007-11-22

**Authors:** Ivan I Senin, Valeriya A Churumova, Pavel P Philippov, Karl-Wilhelm Koch

**Affiliations:** 1 Department of Cell Signalling, A.N. Belozersky Institute of Physico-Chemical Biology, M.V. Lomonosov Moscow State University, 119991 Moscow, Russia; 2 Department of Biology and Environmental Sciences (Biochemistry group), University of Oldenburg, D-26111 Oldenburg, Germany; 3 Research Center Neurosensory Science, University of Oldenburg, D-26111 Oldenburg, Germany; 4 Center of Interface Science, University of Oldenburg, D-26111 Oldenburg, Germany

## Abstract

**Background:**

The Ca^2+^-binding protein recoverin operates as a Ca^2+^-sensor in vertebrate photoreceptor cells. It undergoes a so-called Ca^2+^-myristoyl switch when cytoplasmic Ca^2+^-concentrations fluctuate in the cell. Its covalently attached myristoyl-group is exposed at high Ca^2+^-concentrations and enables recoverin to associate with lipid bilayers and to inhibit its target rhodopsin kinase. At low Ca^2+^-concentrations the myristoyl group is inserted into a hydrophobic pocket of recoverin thereby relieving inhibitory constraint on rhodopsin kinase. Hydrophobic and electrostatic interactions of recoverin with membranes have not been clearly determined, in particular the function of the positively charged carboxy-terminus in recoverin ^191^QKVKEKLKEKKL^202 ^in this context is poorly understood.

**Results:**

Binding of myristoylated recoverin to lipid bilayer depends on the charge distribution in phospholipids. Binding was tested by equilibrium centrifugation and surface plasmon resonance (SPR) assays. It is enhanced to a certain degree by the inclusion of phosphatidylserine (up to 60%) in the lipid mixture. However, a recoverin mutant that lacked the charged carboxy-terminus displayed the same relative binding amplitudes as wildtype (WT) recoverin when bound to neutral or acidic lipids. Instead, the charged carboxy-terminus of recoverin has a significant impact on the biphasic dissociation of recoverin from membranes. On the other hand, the nonmyristoylated WT and truncated mutant form of recoverin did not bind to lipid bilayers to a substantial amount as binding amplitudes observed in SPR measurements are similar to bulk refractive index changes.

**Conclusion:**

Our data indicate a small, but evident electrostatic contribution to the overall binding energy of recoverin association with lipid bilayer. Properties of the charged carboxy-terminus are consistent with a role of this region as an internal effector region that prolongs the time recoverin stays on the membrane by influencing its Ca^2+^-sensitivity.

## Background

A number of proteins with a key function in signal transduction carry covalently attached fatty acid groups. These posttranslational modifications facilitate membrane association, participate in protein translocation from the cytoplasm to membranes or serve as allosteric regulators during conformational changes in proteins [1-4]. A common modification found in many signalling proteins is the myristoyl group, a C14-long fatty acid chain that is covalently attached to an amino-terminal Gly residue. In many proteins, the myristoyl group changes its position or orientation with respect to the polypeptide chain thereby taking part in a myristoyl switch mechanism [2-4]. This switch can be triggered by an external signal as for example phosphorylation, GTP- or Ca^2+^-binding [5]. Systematic studies with short peptides carrying a myristoyl group and comparison with proteins like the tyrosine kinase *src *have revealed that high-affinity association of proteins to membranes (i.e. low dissociation rates) requires a combination of electrostatic and hydrophobic forces [6,7]. While the hydrophobic contribution results from the myristoyl group, the electrostatic driving force comes from an interaction of positive charges in the protein and negative charges of acidic phospholipids in a lipid bilayer. In *src *kinase, a cluster of positively charged amino acids is located within the first 15 amino-terminal amino acids in close proximity to the myristoyl group thereby allowing a high-affinity interaction of src-kinase with phospholipid membranes. Another well studied example is the myristoylatd alanine-rich C kinase substrate called MARCKS that contains a cluster of basic residues separated by 150 amino acids from the Amino-terminal myristoyl group [8-11].

Recoverin, a Ca^2+^-binding protein that belongs to the group of neuronal calcium sensor (NCS) proteins operates as a calcium sensor in photoreceptor cells [12]. It harbours two functional Ca^2+^-binding sites (EF-hand 2 and 3), whereas the first and fourth EF-hand does not bind Ca^2+ ^in micromolar or submicromolar [Ca^2+^]. Recoverin is also myristoylated at its amino-terminus and the Ca^2+^-free (apo) form of recoverin buries the myristoyl tail in a hydrophobic pocket. In contrast, the Ca^2+^-bound form exposes the myristoyl tail into solvent space or into a phospholipid bilayer [13-15]. The sequential binding of two Ca^2+ ^to the third and second EF-hand has been thoroughly investigated [16-18] and several structural studies have contributed to a mechanistic comprehension of the Ca^2+^-myristoyl switch at the molecular level [14,15,19-22]. It was recognized already 10 years ago that recoverin binding to membranes is rather weak in comparison to src-kinase association with membranes [23]. Its apparent K_D _(or EC_50_) for lipid membranes is between between 2.6 × 10^-5 ^and 10^-4 ^M [17,23], which is slightly lower than the Gibbs free binding energy for myristolated glycine (determined to be 10^-4 ^M [6]) and therefore indicates a small, but significant contribution from electrostatic interactions of recoverin with membranes.

Recoverin is lacking a cluster of positive charges at the amino-terminus near the myristoyl tail; instead it harbours a cluster of charged residues at its carboxy-terminus. A solid state NMR study showed that the positive charges at the carboxy-terminus do not interact with phospholipids when myristoylated recoverin is attached to a lipid bilayer [22], but a different conclusion, i.e. participation of the charged carboxy-terminus in the electrostatic interaction of recoverin with membranes, has been drawn from biochemical studies [24]. Recent work using a truncated recoverin form (Rc^2–190^) has demonstrated that a lack of this highly charged protein region changes the Ca^2+^-affinity of recoverin and in consequence other Ca^2+^dependent properties like binding to membranes and inhibition of rhodopsin kinase [25]. Therefore, this region was proposed to act as an internal modulator of Ca^2+^-sensitivity.

The Ca^2+^-dependent binding of recoverin to biological membranes is also influenced by the lipid composition. For example, cholesterol-rich membranes as they are also found in detergent resistant membrane patches from rod outer segment preparations facilitate the membrane association of recoverin [26].

It was the aim of the present work to investigate 1) how the membrane association of recoverin is influenced by the lipid composition 2) how electrostatic interactions contribute eventually to the membrane binding of recoverin and 3) how the positively charged carboxy-terminus of recoverin might influence any membrane binding. We tested the binding of recoverin to different mixtures of lipids, in which the ratio of charged to uncharged molecules was varied. Furthermore, we employed the truncated mutant of recoverin Rc^2–190 ^that lacks the highly charged carboxy-terminus of its WT form for the lipid binding experiments.

## Results

### Hydrophobic and electrostatic interactions contribute to the association of myristoylated recoverin to lipids

**Figure 1:**
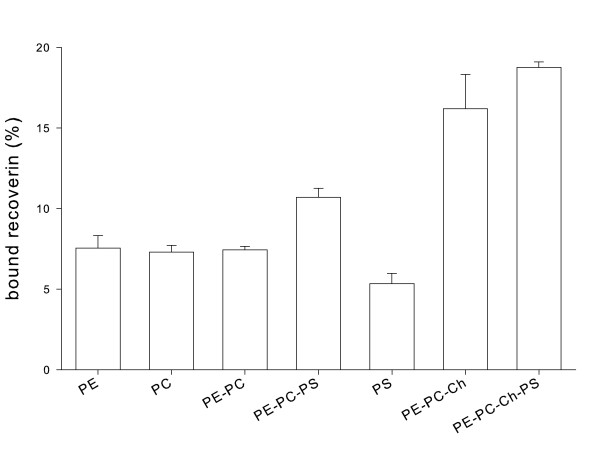
**Binding of myristoylated WT recoverin to different lipids or lipid mixtures**. Binding of recoverin to lipid mixtures was analyzed by an equilibrium centrifugation assay as described in the Methods section. The composition of the lipid mixtures was as follows: PE, 100% (w/w) phosphatidylethanolamine; PC, 100% (w/w) phosphatidylcholine; PE-PC, 50% (w/w) phosphatidylethanolamine, 50% (w/w) phosphatidylcholine; PE-PC-PS, 25% (w/w) phosphatidylethanolamine, 25% (w/w) phosphatidylcholine, 50% (w/w) phosphatidylserine; PS, 100% (w/w) phosphatidylserine; PE-PC-Ch, 25% (w/w) phosphatidylethanolamine, 25% (w/w) phosphatidylcholine, 50%(w/w) cholesterol; PE-PC-Ch-Ps, 20% (w/w) phosphatidylethanolamine, 20% (w/w) phosphatidylcholine, 50% (w/w) cholesterol, 10% (w/w) phosphatidylserine.

**Figure 2:**
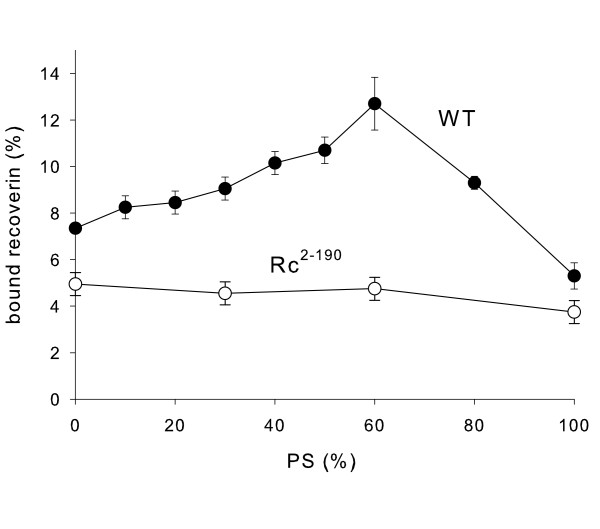
**Binding of myristoylated recoverin to lipid mixtures with different content of PS**. Binding of WT recoverin (filled circles) and Rc^2–190 ^(open circles) to lipid mixtures was analyzed by an equilibrium centrifugation assay as described in the Methods section. The content of PS was adjusted as indicated below. 0% PS: 50% (w/w) phosphatidylethanolamine, 50% (w/w) phosphatidylcholine; 10% PS: 45% (w/w) phosphatidylethanolamine, 45% (w/w) phosphatidylcholine; 20% PS: 40% (w/w) phosphatidylethanolamine, 40% (w/w) phosphatidylcholine; 30% PS, 35% (w/w) phosphatidylethanolamine, 35% (w/w) phosphatidylcholine; 40% PS: 30% (w/w) phosphatidylethanolamine, 30% (w/w) phosphatidylcholine; 50% PS: 25% (w/w) phosphatidylethanolamine, 25% (w/w) phosphatidylcholine; 60% PS: 20% (w/w) phosphatidylethanolamine, 20% (w/w) phosphatidylcholine; 80% PS: 10% (w/w) phosphatidylethanolamine, 10% (w/w) phosphatidylcholine; 100% PS. Ratios of bound WT to bound Rc^2–190 ^were 1.5 (0% PS), 2.0 (30% PS), 2.7 (60% PS) and 1.4 (100% PS).

Liposomes with a different composition of their lipids were made by the extrusion technique and were used in equilibrium centrifugation assays to test binding of myristoylated WT recoverin (Fig. 1). The amount of bound recoverin varied significantly, when different lipid mixtures were compared; the amount was rather low when a pure lipid layer consisting of PE, PC or PE-PC was used. Introduction of a charged phospholipid (PS) into the lipid mixture increased the amount of bound WT recoverin slightly; however, liposomes of pure PS resulted in lower binding as it was observed with PE, PC and PE-PC. In contrast, presence of cholesterol leads to an increase of bound recoverin (two columns on the right site of Fig. 1), which is consistent with previous observations on the effect of cholesterol on membrane binding of recoverin [26]. Negative net charges were implemented into the lipid mixtures by PS. When we increased systematically the percentage of PS in the lipid mixture, the amount of membrane-bound recoverin increased constantly to a maximum at 60% of PS (Fig. 2, filled circles). Further increase of PS leads to a drastic decrease in the amount of bound recoverin. We conclude from these data that a small, but marked electrostatic interaction contributes to the association of myristoylated recoverin with membranes.

### Influence of the highly charged carboxy-terminus of recoverin on its membrane association

The highly charged carboxy-terminus of WT recoverin contains a cluster of 6 Arg residues and 2 Glu residues resulting in a net charge of +4 within this polypeptide region (^191^Q**K**V**K***E***K**L**K***E***KK**L^202^). The truncated mutant of recoverin Rc^2–190 ^lacks this cluster of charges and its three-dimensional structure and some of its biochemcal properties have been previously reported by us [25]. We further investigated how this region contributes to the interaction of recoverin with phospholipids and whether this region is important for the electrostatic effect described above. Therefore, we tested the binding of Rc^2–190 ^to different lipid surfaces in comparison to WT recoverin. Binding of Rc^2–190 ^to lipid mixtures with increasing content of PS was significantly lower in comparison to the binding of WT recoverin to lipids (Fig. 2, open circles). Furthermore, percentage of Rc^2–190 ^binding did not change over the tested range of PS content. These data indicated a significant contribution of the charged carboxy-terminus to the binding of recoverin to lipid mixtures.

**Figure 3:**
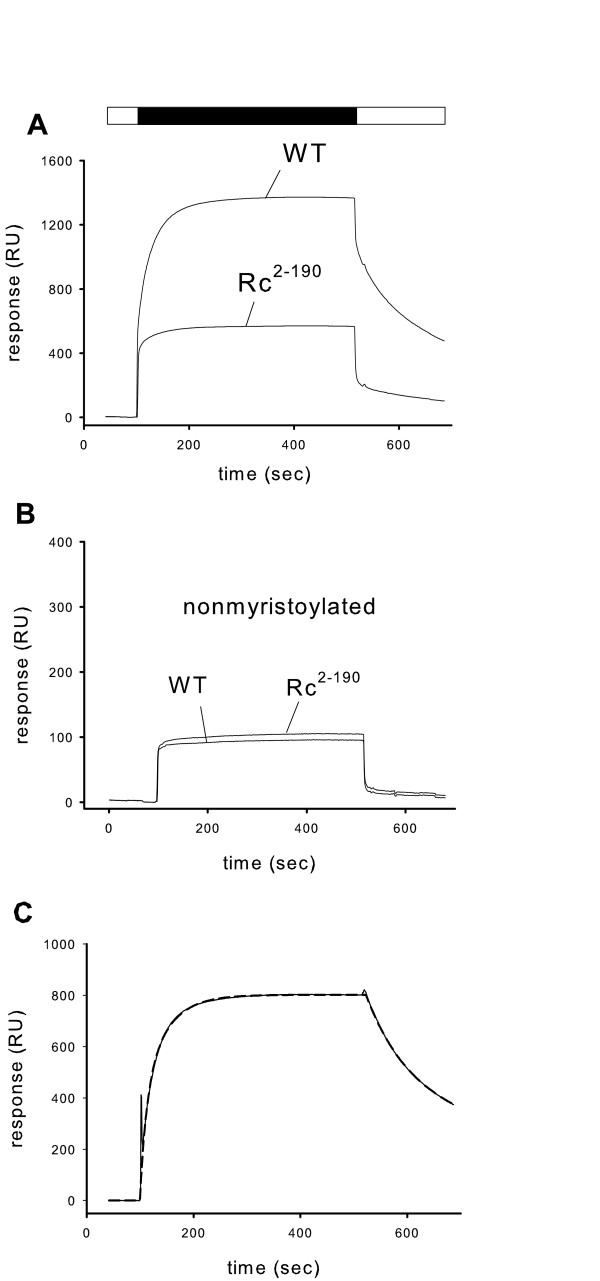
**Surface plasmon resonance analysis of the binding of recoverin forms to an immobilized rod outer segment lipid mixture**. (A) Myristoylated forms of WT recoverin (upper trace) and Rc^2–190 ^were injected at a concentration of 30 μM in SPR running buffer at a flow rate of 5 μl/min. Time of injection is indicated by the black bar at the top. (B) Sensorgrams showing the injection of nonmyristoylated forms of WT recoverin and Rc^2–190 ^into the system. Protein concentration and conditions were the same as in (A). (C) Difference signal (solid curve) obtained by subtracting sensorgram labelled *Rc*^2–190 ^from sensorgram *WT *shown in (A). Dashed curve repesents the fit of the difference curve to the model describing a parallel reaction on a heterogeneous ligand surface (BIAevaluation 4.1). The two corresponding association rate constants were: k_a1 _= 2.29 × 10^3 ^M^-1^s^-1 ^and k_a2 _= 0.707 × 10^3 ^M^-1^s^-1^. Dissociation rate constants were k_d1 _= 0.013 s^-1 ^and k_d2 _= 0.0016 s^-1^.

In order to further investigate this effect we immobilized lipid mixtures on a sensor chip surface and monitored the association and dissociation of proteins by SPR spectroscopy [27,28]. An overlay of typical sensorgrams obtained with the myristoylated forms of recoverin (WT and Rc^2–190^) is shown in Fig. 3a. Composition of the lipid mixture was similar as in rod outer segment membranes and as used previously (40%w/w PE, 40% w/w PC, 15% w/w PS; 5% w/w cholesterol) [17,23]. Sensorgrams for the myristoylated recoverin forms exhibited the typical shapes and amplitudes like we reported previously. Binding amplitude of Rc^2–190 ^was significantly lower than the binding amplitude of WT recoverin. Recently, we attributed this effect to the decrease in Ca^2+^-affinity observed for Rc^2–190 ^(for experimental analysis and discussion on this point please refer to [25]). Nonmyristoylated WT recoverin and Rc^2–190 ^bound to the same surface with much lower, but equal amplitudes (note different scaling in Fig. 3b). However, amplitudes resulted mainly from a bulk refractive index change and did not represent a Ca^2+^-dependent binding of recoverin forms to immobilized lipids [17].

### Binding of WT recoverin and Rc^2–190 ^to different lipid surfaces

**Figure 4:**
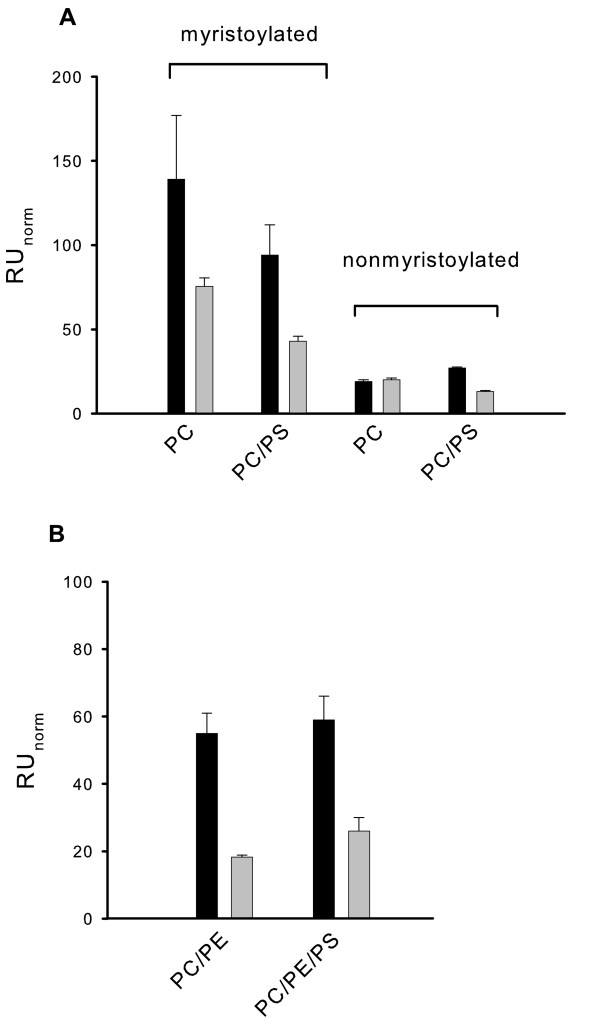
**Normalized amplitudes of SPR sensorgrams obtained by injection of recoverin forms into flow cells coated with different lipid mixtures**. (A) Binding of myristoylated and nonmyristoylated forms of both WT recoverin (black bars) and Rc^2–190 ^(gray bars) were compared by employing a pure PC and a PC:PS (50:50) surface. Protein concentration was 30 μM. Estimated ratios WT/Rc^2–190 ^of binding amplitudes were 1.8 (myristoylated, PC), 2.2 (myristoylated, PC/PS), 0.9 (nonmyristoylated, PC) and 2.0 (nonmyristoylated, PC/PS). (B) Binding of myristoylated WT recoverin (black bars) and Rc^2–190 ^(gray bars) on a PC:PE (50:50) or a PC:PE:PS (40:40:20) surface yielding ratios of 3.0 and 2.3, respectively. Protein concentration was 5 μM. Data were collected from 2–6 experiments.

In order to test the influence of different lipids on the membrane association of recoverin, we immobilized different lipid mixtures on the sensor chip surface and measured the binding amplitudes for myristoylated and nonmyristoylated forms of both WT recoverin and Rc^2–190^. Normalized amplitudes obtained with myristoylated WT and Rc^2–190 ^on pure PC or on a PC/PS mixture resulted in different amplitudes (Fig. 4a); however, the ratio of WT/Rc^2–190 ^amplitudes measured with PS containing lipids was like in pure PC (s. legends to Fig. 4). A similar observation was made with lipid mixtures containing PC/PE and PC/PE/PS (Fig. 4b). Taking the results of Fig. 2 into account one could conclude that the carboxy-terminus facilitates binding to lipid mixtures containing a certain degree of negative net charges. However, the ratios of bound WT to bound Rc^2–190 ^were between 1.4 and 2.7 and thus were even below the ratio of 3.0 obtained with a PC:PE surface (Fig. 4b). We conclude from these results that the charged cluster in the carboxy-terminus of recoverin is not involved in the electrostatic contribution of recoverin's interaction with lipid membranes.

**Figure 5:**
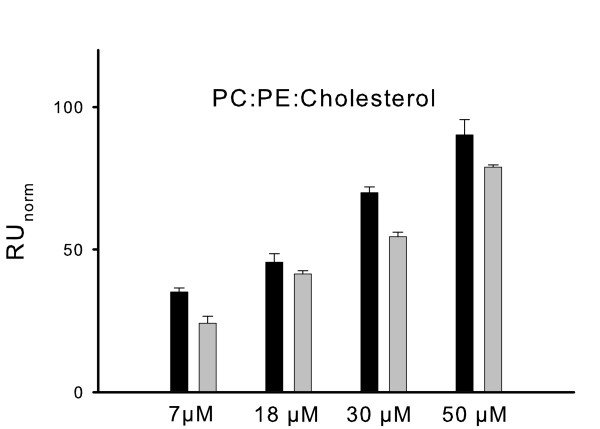
**Normalized amplitudes of SPR sensorgrams obtained by injection of recoverin forms into flow cells coated with PC:PE:cholesterol**. Injection of WT recoverin (black bars) and Rc^2–190 ^(gray bars) into the flow cell at the indicated concentrations. The immobilized lipid surface contained a mixture of PC:PE:cholesterol (25:25:50). The binding ratios were 1.45 (7 μM), 1.1 (18 μM), 1.28 (30 μM) and 1.14 (μM). Data shown were collected from 2 experiments and are representative for a different set of experiments with cholesterol containing lipids. The running buffer containing 0.2 mM CaCl_2 _in all cases.

Sensorgrams that were recorded with nonmyristoylated recoverin forms on PC and PC/PS surfaces were similar in shape and amplitudes as shown in Fig. 3b and yielded very low normalized RU values (right part of Fig. 4a). Although some difference was seen between nonmyristoylated WT and Rc^2–190 ^on a PC/PS lipid mixture (Fig. 4a), the normalized RU values fall within a range that is typical for bulk refractive index changes of SPR recordings [17] and are therefore not considered for further analysis. Cholesterol had a more pronounced effect on the membrane binding of WT recoverin than PS containing lipid mixtures had (Fig. 1). Using a PC: PE: cholesterol mixture as lipid surface (Fig. 5), we compared the binding amplitudes of WT recoverin with the amplitudes of Rc^2–190^. Amplitudes increased with increasing amounts of recoverin forms (7 – 50 μM). Normalized amplitudes of WT recoverin were in all cases higher than amplitudes obtained with Rc^2–190^, but the WT/Rc^2–190 ^ratio was significantly lower than it was observed with cholesterol-free lipids (Fig. 4 and 5). We conclude from these data that cholesterol facilitates binding of Rc^2–190 ^to lipids and its presence could partially compensate for the lack of the charged carboxy-terminus.

### Kinetic analysis of SPR binding signals

SPR sensorgrams that were recorded with myristoylated WT recoverin exhibited biphasic association and dissociation kinetics consisting of a slow and a fast phase [17,23]. This can be seen for instance with WT recoverin in Fig. 3a. However, the SPR signals that were recorded with Rc^2–190 ^did never exhibit a prominent slow phase (Fig. 3a). Instead the signal consisted mainly of a fast association and dissociation phase. In order to gain more information to which extent the charged carboxy-terminus contributes to the slow phase, we subtracted the Rc^2–190 ^signal from the WT signal in Fig. 3a which gave the difference trace shown in Fig. 3c. If the slow phase was solely determined by the presence of the charged carboxy-terminus, the signal should have become monophasic. However, the difference signal still consisted of a fast and slow phase and we were unable to fit it to a monoexponential Langmuir binding model (BIAevaluation 4.1.). The best fit of the sensorgram in Fig. 3c was obtained with a model assuming a heterogeneous ligand and a parallel reaction (dashed curve in Fig. 3c). When we applied the subtraction procedure to other sets of sensorgrams (e.g. lipid mixtures as used for the experiments in Fig. 4), the signals did not become monophasic. This indicates that the charged carboxy-terminus contributes significantly to the slow phase of the sensorgrams usually recorded with WT recoverin, but apparently it is not the sole determinant of this kinetic feature.

## Discussion

Myristoyl switches trigger reversible protein-membrane interactions and represent a mechanism to control protein translocation from the cytoplasm to cell membranes and vice versa. The reversible association of proteins to membranes can be controlled by hydrophobic as well as electrostatic interactions [2,4]. We report here experimental evidence that the lipid composition had a significant effect on the total amount of recoverin bound to membranes (Fig. 1 and 2), but the most important molecular component for membrane association is the myristoyl group (Fig. 3), because nonmyristoylated forms of WT recoverin and the mutant Rc^2–190 ^displayed the same low binding amplitudes that are almost indentical to the bulk refractive index changes typically for SPR experiments (see ref. [17] for a thorough discussion of this point). These results are consistent with our and other previous reports that analyse recoverin binding to lipid bilayers by SPR spectroscopy [17,23] and atomic force microscopy technology [29], but they disagree with a recent publication by Desmeules et al., who used monolayers and polarization modulation infrared reflection absorption spectroscopy in their analysis [30]. These authors found that myristoylated and nonmyristoylated recoverin exhibit significant binding to monolayers in the presence and absence of Ca^2+^, however with slower binding kinetics of the Ca^2+^-free forms. We have no explanation for the differences except that lipid monolayers and not lipid bilayers were used in the recent study. We can exclude that the SPR studies are limited by the time course of the recording. Injection of proteins into the flow cell and the buffer flow can be controlled over a range of 1–100 μl/min resulting in different time intervals for recording. Sensorgrams in Fig. 3 were recorded over a time interval of 600 sec with a flow rate of 5 μl/min. All recordings showed that the binding process reached equilibrium over the time course of protein injection (see plateau phase in Fig. 3). Thus, slower binding events should have been detected.

Although the highly charged carboxy-terminus of recoverin is not directly involved in the electrostatic contribution of the binding energy, it apparently plays an important modulatory role for the binding mechanism in general. Myristoylated WT recoverin displayed a complex biphasic association and dissociation phase consisting of a fast and slow phases (Fig. 3) that had been analyzed by us in previous work [17,23]. Binding of the truncated myristoylated recoverin mutant Rc^2–190 ^to mixed ROS lipid bilayers occurred with fast association and dissociation kinetics. One could suggest from the sensorgrams in Fig. 3a that the charged carboxy-terminus is the molecular determinant of the slow phase. Therefore, we subtracted the Rc^2–190 ^sensorgram from the WT sensorgram yielding the difference signal in Fig. 3c, where the dissociation phase appeared much slower. However, fitting of the difference signal to a monoexponential Langmuir binding model gave unsatisfying results, instead the best fit was obtained with a parallel binding model assuming a heterogeneous surface (BIAevaluation 4.1). It is still an unresolved issue whether liposomes when injected into the Biacore system fuse with the L1 sensor chip and form a bilayer or whether they are mainly immobilized as intact spheres (see for example discussion in [28]). Thus, we interpret our result as that recoverin binds with different kinetics to immobilized lipids due to their inherent heterogeneity.

However, the subtraction procedure allows highlighting that the slower dissociation phase originates from the charged carboxy-terminus. The charged carboxy-terminus would therefore represent an effector region in recoverin, which is consistent with our recent assumption that the charged carboxy-terminus operates as an internal modulator of Ca^2+^-sensitivity [26]. Combining both observations (this work and [26]) we conclude that the carboxy-terminus of recoverin is an effector region that delays the dissociation of recoverin from the membrane by influencing its Ca^2+^-sensitivity. A further consequence of this conclusion is that the recoverin target rhodopsin kinase would be inhibited less efficiently by Rc^2–190 ^in comparison to WT recoverin, because recoverin would have a reduced mean residence time at the membrane. Indeed, we reported previously [26] that inhibition of rhodopsin kinase by Rc^2–190 ^is shifted to higher free Ca^2+^-concentration and thereby the inhibition becomes less efficient.

Clusters with a high number of positively charged regions are also found in recoverin orthologs, but not in other NCS proteins like neurocalcin, VILIP and NCS-1 (see for example discussion in [24]). Isoforms of another subgroup of NCS proteins called guanylate cyclase-activating proteins (GCAPs) display rather diverse carboxy-termini with a low degree of sequence homology, but at least two isoforms, GCAP2 and GCAP3 [31-33] have a cluster of positive charges. For example, bovine GCAP2 harbours a region with a net charge of +3 (^198^**RRK**SAMF^204^) and zebrafish GCAP3 contains a region with a net charge of +3 (^181^NGQ**KKKK***E*^188^). Human GCAP3 has a more extended carboxy-terminus with a net charge of +4 [33]. It remains to be shown, whether these regions in GCAPs operate as an internal modulator of their Ca^2+^-sensitivity or participate in electrostatic interactions with membranes or target proteins.

## Conclusion

Myristoylated recoverin exhibits a transient association with biological membranes that is controlled by hydrophobic and electrostatic interactions. The main driving force of this process is the insertion of the myristoyl group into the lipid bilayer. Electrostatic interactions do not origin from the highly charged carboxy-terminus, although this region of recoverin is important for its membrane association in general. It represents an internal effector region that might prolong the time myristoylated recoverin is associated with membranes. Thereby it ensures sufficient interaction between recoverin and its target rhodopsin kinase.

## Methods

### Heterologous Expression and Purification of Proteins

The expression construct for the truncated bovine recoverin mutant Rc^2–190 ^was generated by site-directed mutagenesis and the correct DNA sequence was verified by sequencing as described in detail previously [25]. Heterologous expression and purification of all myristoylated and non-myristoylated recoverin forms was performed and analyzed as described previously [17,25]. Degree of myristoylation was checked by reversed phase high performance liquid chromatography using a Vydac 238TP C18 column (4.6 × 250 mm). Usually more than 90% of purified recoverin forms were myristoylated when coexpressed with yeast N-myristoyl-transferase (kindly provided by Dr. Jeffrey Gordon, Department of Molecular Biology and Pharmacology, Washington University School of Medicine, St. Louis). In cases where the amount of myristoylated protein was not sufficient, recoverin forms were further purified on the same reversed phase column and denatured protein was refolded as described [17].

### Binding of Recoverin Forms to Membranes

Liposomes were prepared from phosphatidylcholine (PC), phosphatidylethanolamine (PE), phosphatidylserine (PS) and/or cholesterol by the extrusion technique essentiall as described before [17,23]. The percentage amount of each lipid was adjusted in the mixture as needed for the specific experiment. For example, a mixture of lipids containing 40% (w/w) PE, 40% (w/w) PC, 15% (w/w) PS; 5% (w/w) cholesterol corresponds to the lipid composition in bovine rod outer segment membranes. Typically, a mixture of 4 mg of lipids of desired composition were mixed and dried down by vacuum in a SpeedVac concentrator. The sample was resuspended in 2 ml of degassed buffer (20 mM HEPES, pH 7.5,150 mM NaCl) and sonified for 2 × 15 min (Branson B12; cup; 100 W). Large unilamellar vesicles(liposomes) were produced using the extrusion technique. The suspensionwas soaked for 15–20 min and extruded through polycarbonate filterwith a pore diameter of 1 μm (first step) and 0.4 μm (second step). The extrusion technique yielded a homogeneous suspension of vesicles with a size of approximately 100 nm as determined by dynamic light scattering [34]. Liposomes (1 mg/ml) were added to purified myristoylated recoverin forms (30 μM) and incubated for 30 min at 37°C (Eppendorf Thermomixer 5436,1000 rpm) for 15 min in 20 mM HEPES, pH 7.5,150 mM NaCl, 20 mM MgCl_2_, 1 mM DTT, 3 mM dibromo-BAPTA, and 20 mM CaCl_2 _(totalvolume 75 μl). Vesicles were separated by centrifugation (15 min, 14,000 rpm; Eppendorf model 5415 tabletop centrifuge) and the supernatant was removed. The amount of bound recoverin was determined by densitometry of Coomassie Blue stained sodium dodecylsulfate polyacrylamide gels [17].

### Surface Plasmon Resonance Measurements

In a second approach to test and quantify the binding of recoverin to lipids we employed SPR spectrocopy [17,23,27,28]. Lipid mixtures were immobilized on commercially available sensor chips (L1, BIAcore) and recoverin forms were injected into the mobile phase (running buffer consisted of 10 mM Hepes, pH 7.5, 150 mM KCl, 20 mM MgCl_2 _and 0.4 mM CaCl_2_) and flushed over the flow cell. Binding events are seen as a positive change in resonance units (RU) and were recorded as a function of time to obtain sensorgrams. In order to account for variations in the immobilization densities of lipids, we normalized the amplitudes of sensorgrams to the amount of lipids on the sensor chip surface. Details of SPR operation, of control recordings and evaluation procedures have been described before [17,23]. Data processing was performed with the BIAevaluation 4.1 software.

## List of abbreviations used

PC, phosphatidylcholine; PE, phosphatidylethanolamine; PS, phosphatidylserine; WT, wildtype; Rc^2–190^, truncated recoverin mutant, SPR, surface plasmon resonance

## Authors' contributions

IIS carried out the lipid binding experiments and the SPR studies. VAC carried out mutant preparation, its expression and purification. IIS and KWK analyzed the data. All authors conceived of the study and participated in its design. KWK wrote the first draft of the manuscript, IIS and PPP helped to draft the manuscript. All authors read and approved the final manuscript.
